# The bromodomain and extraterminal domain inhibitor bromosporine synergistically reactivates latent HIV-1 in latently infected cells

**DOI:** 10.18632/oncotarget.21585

**Published:** 2017-10-06

**Authors:** Hanyu Pan, Panpan Lu, Yinzhong Shen, Yanan Wang, Zhengtao Jiang, Xinyi Yang, Yangcheng Zhong, He Yang, Inam Ulla Khan, Muya Zhou, Bokang Li, Ziyu Zhang, Jianqing Xu, Hongzhou Lu, Huanzhang Zhu

**Affiliations:** ^1^ State Key Laboratory of Genetic Engineering and Key Laboratory of Medical Molecular Virology of Ministry of Education/Health, School of Life Sciences, Fudan University, Shanghai, China; ^2^ Department of Infectious Diseases and Key Laboratory of Medical Molecular Virology of Ministry of Education/Health, Shanghai Public Health Clinical Center, Fudan University, Shanghai, China

**Keywords:** bromosporine, HIV-1 latency, reactivation, BET inhibitor, CDK9 T-loop

## Abstract

The long-lived latent HIV-1 reservoir is the major barrier for complete cure of Acquired Immune Deficiency Syndrome (AIDS). Here we report that a novel bromodomain and extraterminal domain (BET) inhibitor bromosporine which can broadly target BETs, is able to potently reactivate HIV-1 replication in different latency models alone and more powerful when combined with prostratin or TNF-α. Furthermore, the treatment with bromosporine induced HIV-1 full-length transcripts in resting CD4+ T cells from infected individuals with suppressive antiretroviral therapy (ART) *ex vivo*, with no obvious cytotoxicity or global activation of T cell. Finally, our data suggest that Tat plays a critical role in the bromosporine-mediated reactivation of latent HIV-1, which involved the increase of CDK9 T-loop phosphorylation. In summary, we found that the BET inhibitor bromosporine, alone or with other activators, might be a candidate for future HIV-1 eradication strategies.

## INTRODUCTION

Since the recognition of Acquired Immunodeficiency Syndrome (AIDS) in 1981 [1], there is still no winning strategy to completely cure AIDS. According to the report of world health organization (WHO) in 2015, there were approximately 36.7 million people living with HIV-1 (Human Immunodeficiency Virus type 1), the causative agent of the AIDS, except for those who have died of AIDS. Despite the success in suppressing HIV-1 to undetectable levels by ART [2], the latent reservoirs of integrated HIV-1 proviruses (mainly in resting CD4+ T cells) still remain to be the major hurdle to virus eradication, which makes the virus rapidly rebound to pretreatment levels once interrupting ART [3, 4], since most cells in the reservoir are hidden from immune surveillance. Due to the tremendous size of latent reservoirs, which are likely established within days of infection [5], estimated to be 10^5^–10^6^ cells per patient, it will take more than 70 years to eradicate HIV-1 if just treated with ART [6, 7]. In addition, HIV is also associated with complications like a higher than normal risk of cardiovascular disease, cancer, osteoporosis and other end-organ diseases [2]. Therefore, elimination of the latent reservoirs of HIV is extremely important to overcome AIDS completely.

The “Shock and Kill” strategy [8] is one of the strategies that have gained much attention to eradicate latent HIV-1 reservoirs. This strategy operates to reactivate latent reservoirs followed by eliminating them through ART, neutralizing antibodies or/and other agents. With the progress of findings in the molecular mechanisms underlying HIV-1 proviral latency [9], more and more efficient small molecules have been shown to be useful for the first step “Shock” to stimulate HIV-1 transcription in latently infected cells [10], including several main groups: DNA methyltransferase inhibitors, histone deacetylase inhibitors (HDACis), histone methyltransferase (HMT) inhibitors, positive transcription elongation factor b (P-TEFb) activators and protein kinase C (PKC) activators. Some agents among them even have been investigated in clinical trials like Vorinostat (SAHA) [11]. Though these compounds did reactivate latent HIV-1, results showed that none of them was both safe and effective in trials involving enlarged sample size and prolonged treatment [12–16]. Due to the disadvantages of these drugs, seeking for new safer and more effective HIV-1 activators to induce virus production without causing global T cell activation has been the “research priority” these years [17].

Recently, many researchers have been paying attention to the pharmacologic inhibition aimed at members of the bromodomain and extraterminal domain (BET) family as a therapy strategy. As a well-conserved family of transcriptional regulators, the structure of BET proteins involves tandem bromodomains, conserved domains and an extraterminal domain [18, 19]. The first inhibitors developed to target BET proteins showed potent activity in cancers of diverse types [20]. As for HIV-1, targeting the binding of BET proteins to chromatin was also reported to be important for the regulation of HIV-1 gene expression, especially transcription elongation [21]. JQ1, one of the BET inhibitors, has successfully reactivated HIV-1 in different latency models and in ART treated patients combined with an HDACi or PKC agonist [21–24]. Another compound of the BET inhibitors called OTX015, was also proved to be suitable for human use [25] and able to reverse HIV-1 latency *in vitro* either alone or combined with other agents [26]. A leading compound from a set of UMB-32 analogs, UMB136, was also demonstrated to be potent in reactivating latent HIV-1 as a BET inhibitor in different cell line models and *ex vivo* models [27]. Recently, a novel promiscuous BET inhibitor bromosporine was developed that broadly targeted BETs [20] and has gained much attention. Here, we examined whether bromosporine could influence the latency of HIV-1. Results indicate that bromosporine can potently reactivate HIV-1 replication from latency through an increase of CDK9 T-loop phosphorylation in HIV-1 latency models *in vitro*. Also, combining bromosporine with some other activators could obviously augment the effect than using anyone among them alone. Furthermore, the treatment with bromosporine also induced the expression of latent HIV-1 in primary CD4+ T cells from individuals having suppressive ART *ex vivo* with no distinct toxicity or global activation of T cell.

## RESULTS

### Bromosporine reactivates HIV-1 replication *in vitro* in latent HIV-1 cell lines

**Figure 1:**
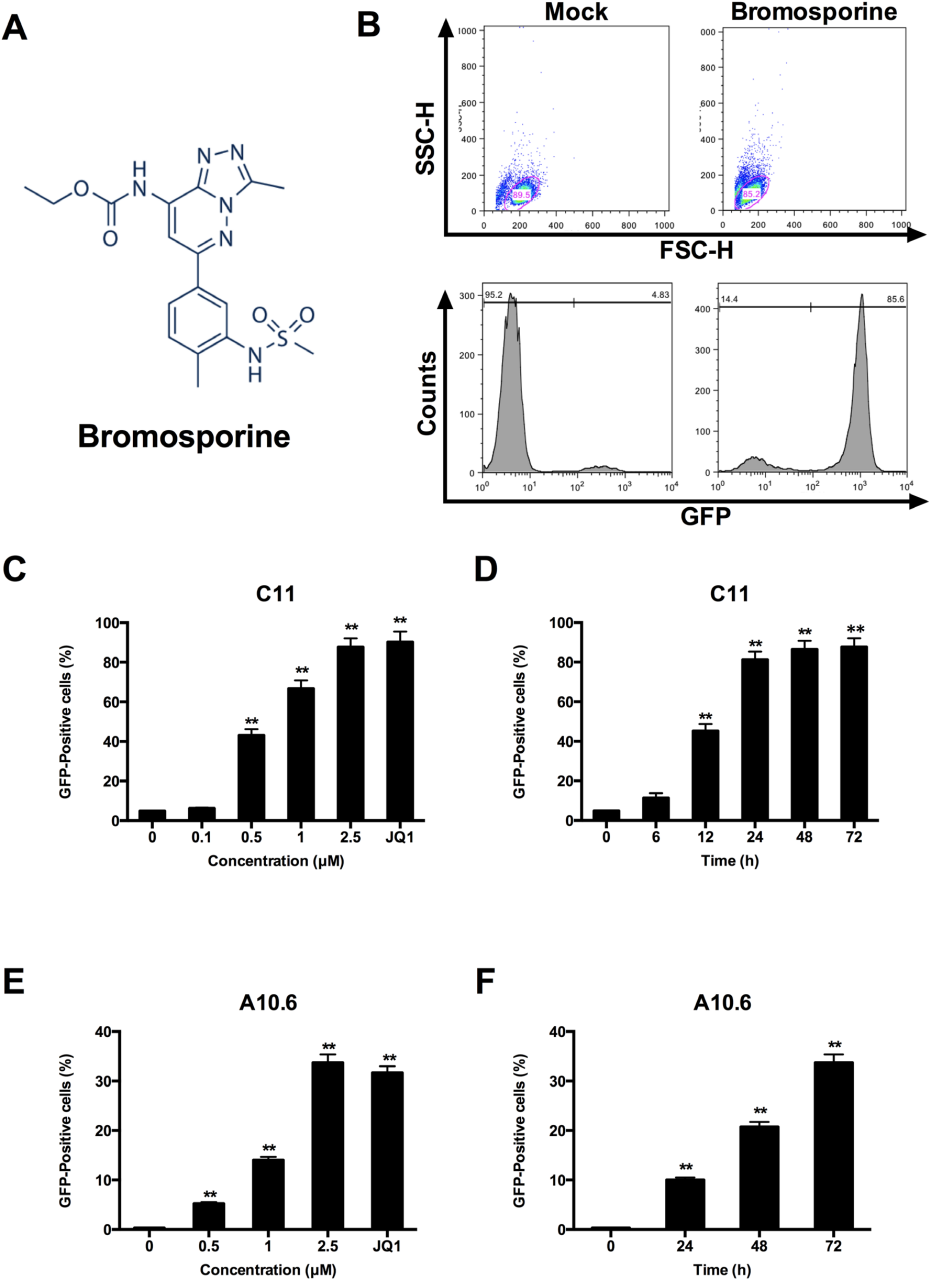
Bromosporine activates HIV-1 replication *in vitro* in latent HIV-1 cell culture models **(A)** The structure of bromosporine. **(B)** J-Lat clone C11 cells were treated with 2.5 μM bromosporine for 72h and induction of GFP, representing the level of HIV-1 transcription, was measured by flow cytometry and presented as fluorescence histograms. **(C)** C11 cells were treated with bromosporine for 72h at the indicated concentrations or treated with JQ1 (1 μM) for 72h. Results are expressed as a percentage of GFP-positive cells within the entire population. **(D)** C11 cells were mock-treated or treated with 2.5 μM bromosporine for the indicated time period, and the results are expressed as percentage of GFP-positive cells in the entire population. **(E, F)** A10.6 cells were treated and analyzed as in (C, D). ^*^p < 0.05, ^**^p < 0.01.

The chemical structure of bromosporine is shown in Figure 1A. To evaluate the potential of bromosporine to induce HIV-1 expression in latently infected cells, we used C11 cell line, a clonal which had been previously raised in our laboratory [28]. The C11 cells were Jurkat cells latently infected with a single provirus integrated into intron of RNPS1 and encoding the green florescence protein (GFP) under the control of HIV-1 LTR as a marker of HIV-1 expression. After treating with 2.5 μM bromosporine for 72h, the percentage of GFP-expressing cells was measured by flow cytometry, which represented the expression of HIV-1 LTR-driven GFP. The percentage of GFP-positive cells increased to 85.6% as compared to mock treatment (Figure 1B). In addition, dose- and time-dependent effects of bromosporine on HIV-1 reactivation were also observed in C11 cells (Figure 1C and 1D) (Supplementary Figure 1). As shown in Figure 1C, the percentage of GFP-positive cells dramatically raised from 6.88% to 87.7% as the concentration of bromosporine increased from 0.1 μM to 2.5 μM. And as shown in Figure 1D, after C11 cells were treated with 2.5 μM bromosporine, the percentage of GFP-positive cells increased as a function of time.

J-Lat clone A10.6 cells, which is also a Jurkat T cell line latently infected by HIV-1 [29, 30], were further used in order to examine whether similar results could be obtained in other latently infected T cells. Results from these cells also indicated that bromosporine can potently reactivate latent HIV-1 replication in a dose- and time-dependent manner (Figure 1E and 1F) (Supplementary Figure 2). In conclusion, the data presented above show the powerful ability of bromosporine in reactivating latent HIV-1 in different latently infected Jurkat T cell models.

### Synergistic reactivation of HIV-1 by bromosporine and other activators in latently infected cells

**Figure 2:**
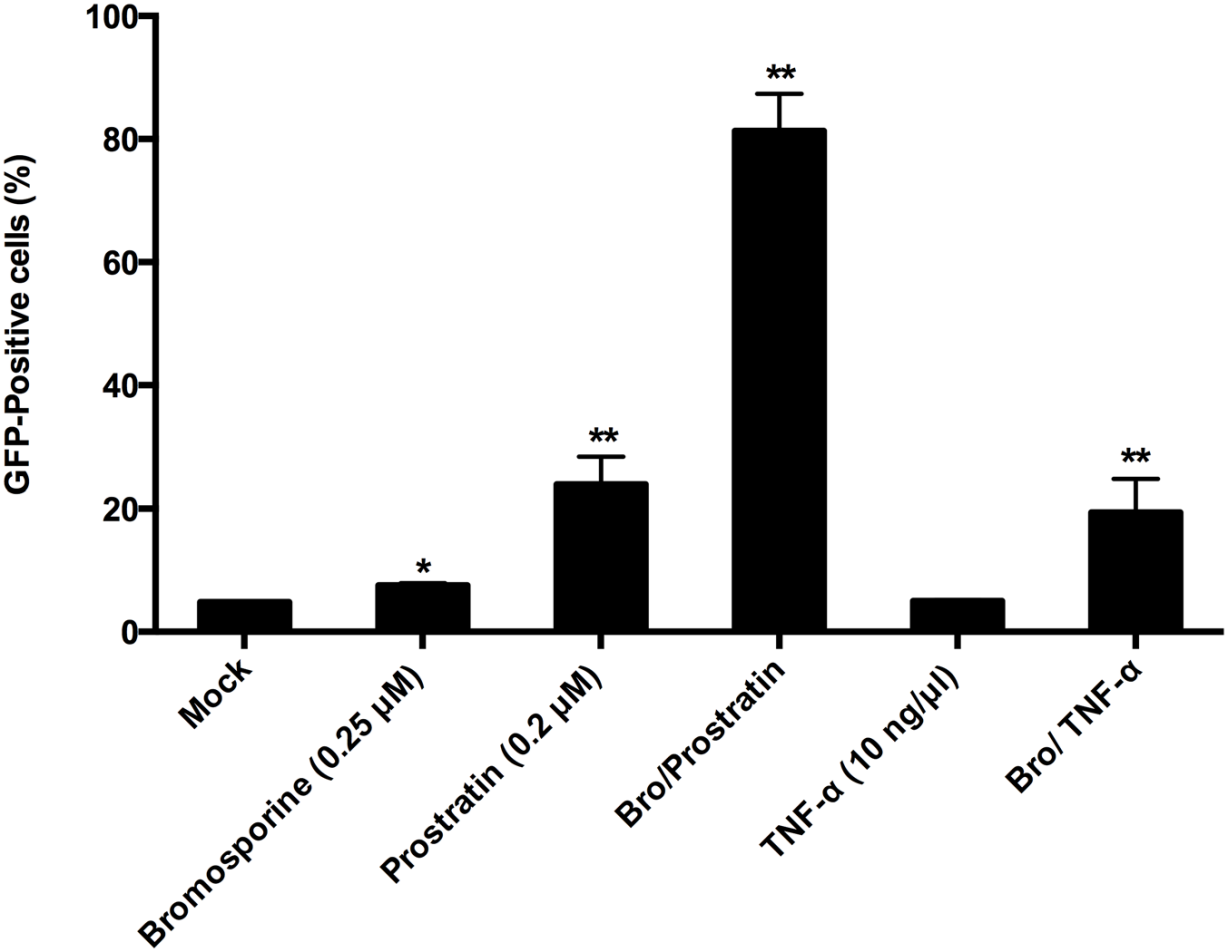
Synergistic reactivation of HIV-1 promoter by bromosporine and prostratin, TNF-α in latently infected cells C11 cells were mock-treated or treated with either bromosporine (0.25 μM), prostratin (0.2 μM), TNF-α (10 ng/μl) or bromosporine (0.25 μM)/prostratin (0.2 μM), bromosporine (0.25 μM)/ TNF-α (10 ng/μl). The effect of activation of the HIV-1 promoter was determined by quantifying GFP-positive cells 72h after treatment using flow cytometry. A summary of the activation assays is presented as a series of histograms. ^*^p < 0.05, ^**^p < 0.01.

The establishment and maintenance of HIV-1 latency underlies multiple signaling pathways and molecular mechanisms [8, 9, 31], so we utilized prostratin or TNF-α in combination with bromosporine in order to investigate whether bromosporine synergistically reactivates the HIV-1 promoter. C11 cells were mock treated or treated with bromosporine (0.25 μM), prostratin (0.2 μM), TNF-α (10 ng/μl), bromosporine (0.25 μM)/prostratin (0.2 μM), or bromosporine (0.25 μM)/ TNF-α (10 ng/μl) for 72h, respectively. We used a lower concentration here due to the high potency of bromosporine in reactivating latent HIV-1 replication which would make it difficult to distinguish the combined effects of bromosporine with other activators at higher concentrations. As shown in Figure 2 and Supplementary Figure 3, when cells were treated with bromosporine, prostratin or TNF-α alone, the percentage of GFP-expressing cells in each group was only about 7.54%, 23.9% and 4.99%, respectively. However, when cells were co-treated with bromosporine and prostratin or bromosporine and TNF-α, the percentage of GFP-positive C11 cells raised markedly.

To further evaluate the synergic activity of bromosporine combined with prostratin or TNF-α, we used the Bliss independence model [32] as a metric and compared the predicted effect with experimentally observed effect of the combined drugs. This model assumes that the effects of two compounds which act through different mechanisms are merely additive in the absence of synergistic interactions. In contrast, the effects of drug combinations greater or lesser than the ideal Bliss independence prediction imply synergy or antagonism, respectively. As shown in Figure 2 and Supplementary Figure 3, when cells were co-treated with bromosporine and prostratin or bromosporine and TNF-α, the percentage of GFP-positive C11 cells over and above the DMSO control was 76.5% and 14.6%, respectively, which was much higher than the prediction (21.3% and 2.87%, respectively) calculated according to Materials and Methods description. These results show that bromosporine potentially synergize with prostratin or TNF-α to reactivate HIV-1 from latency in C11 cell line.

### Bromosporine reactivates HIV-1 replication from latency in primary CD4+ T cells from individuals with suppressive ART *ex vivo*

**Figure 3:**
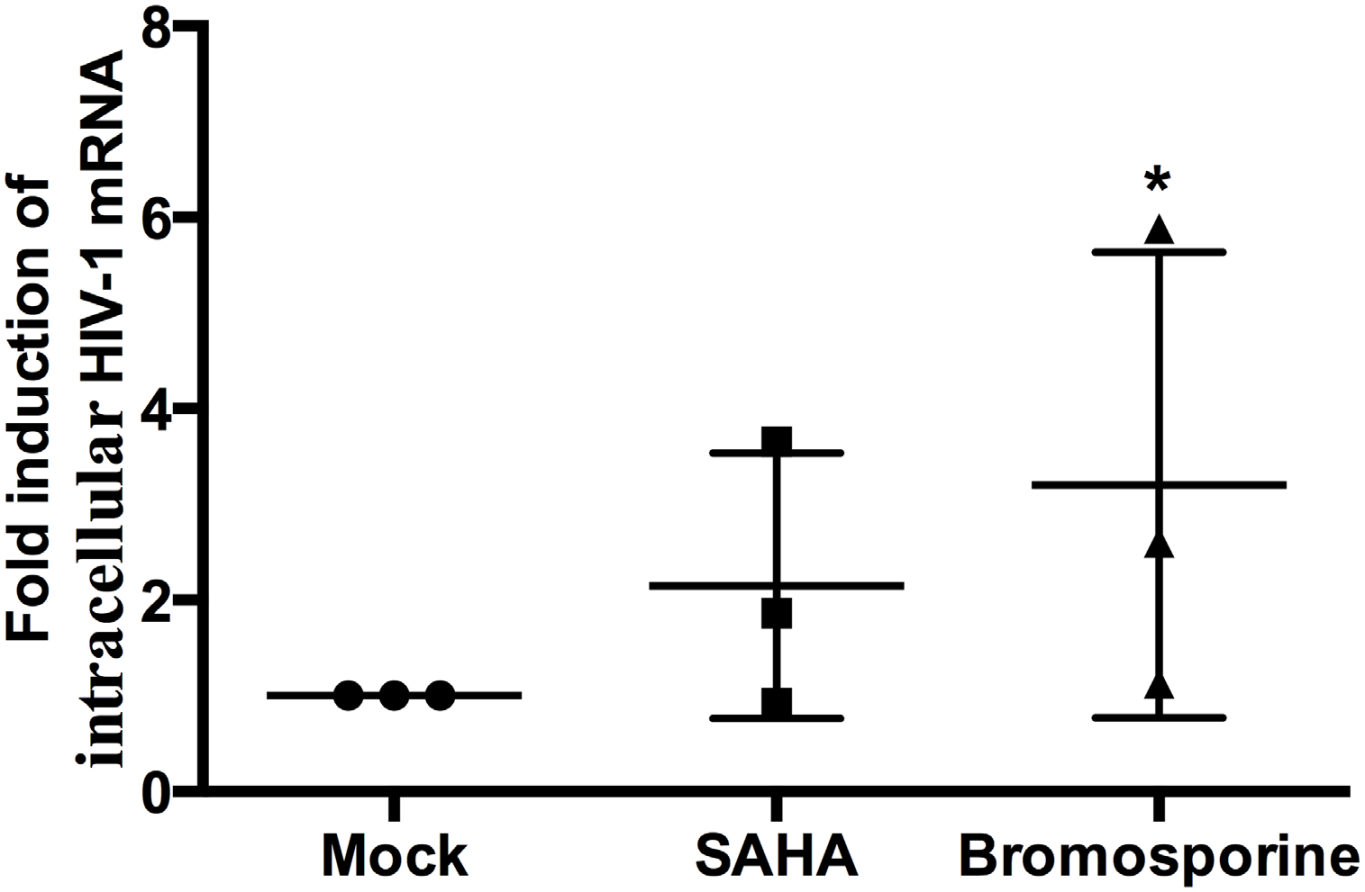
Bromosporine reactivates HIV-1 replication from latency in primary CD4+ T cells from individuals with suppressive ART *ex vivo* Resting CD4+ T cells from three infected individuals were treated with 2.5 μM bromosporine or 500 nM SAHA for 18h. Intracellular HIV-1 mRNA levels were measured by real-time PCR for the Poly A region and presented as fold induction relative to the DMSO control. ^*^p < 0.05.

In order to measure the ability of bromosporine to reactivate HIV-1 replication from latency in resting CD4+ T cells, we first purified the resting CD4+ T cells from the peripheral blood of HIV-1 infected individuals with suppressive ART and incubated with 2.5 μM bromosporine or 500 nM suberoylanilide hydroxamic acid (SAHA) for 18h. We used real-time PCR with primers/probe specific for the HIV-1 3’ poly A region to measure intracellular HIV-1 mRNA expression levels as described earlier [12]. Despite the innate difference of fold induction of HIV-1 transcription between donors resulting from the connaturally variable response of latently infected resting CD4+ T cells to activators, bromosporine induced an obvious (>2-fold) increase in HIV-1 transcription in two of the three donors. As to the cells treated with SAHA, only one donor showed >2-fold increase (Figure 3). These results reveal that bromosporine is potent in reactivating HIV-1 from latently infected primary CD4+ T cells *ex vivo*.

### Bromosporine does not induce marked toxicity in primary CD4+ T cells

**Figure 4:**
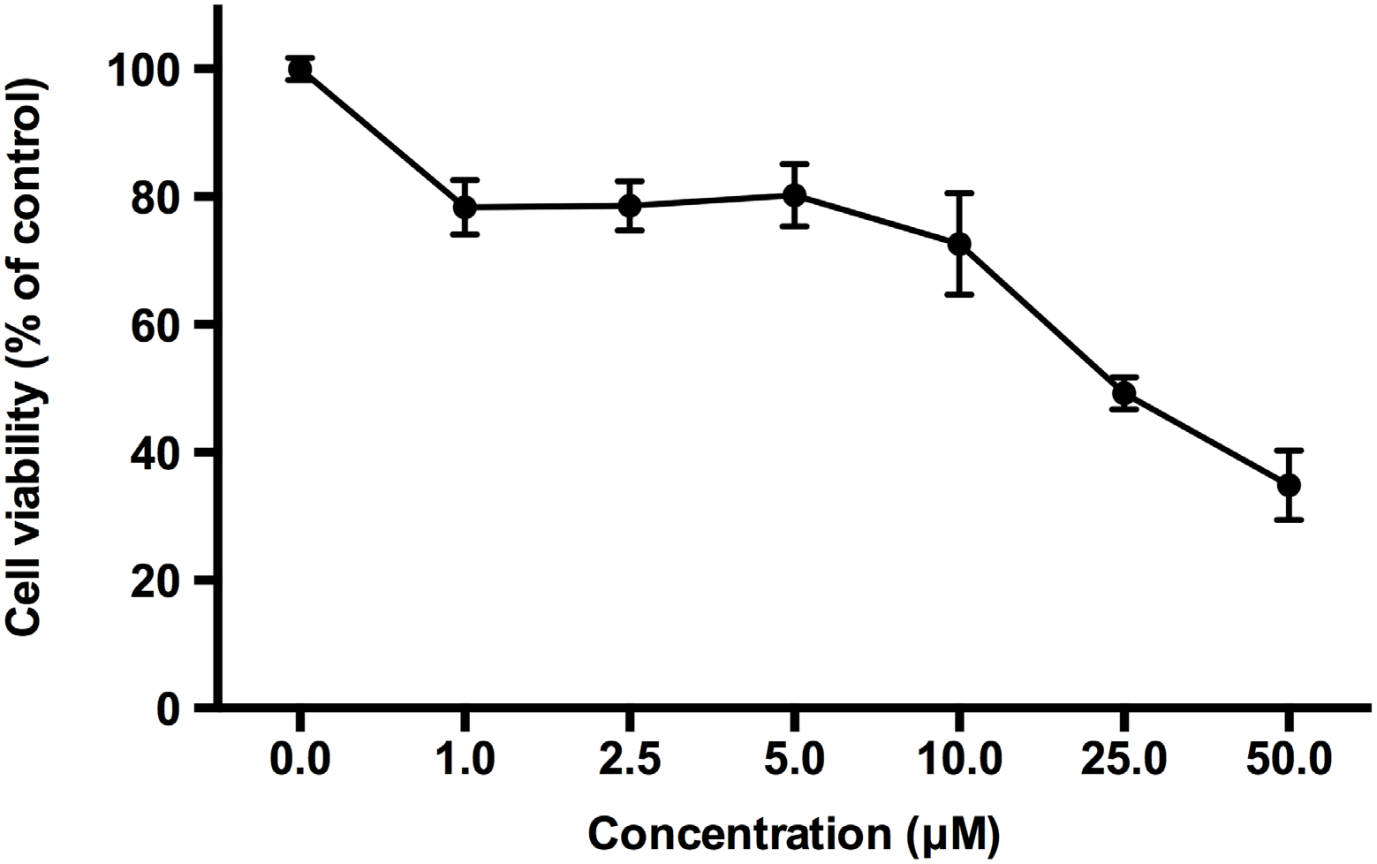
The effects of bromosporine on cell viability in PBMCs are non-significant at its active concentration PBMCs from HIV-negative donors were treated with bromosporine at the indicated concentrations for 72h and then cell viability was measured by CCK-8 kit (Dojindo). The division of OD450 between groups treated with different drug concentrations indicated the percentage of cell viability.

Low toxicity is an essential property of an ideal activator for clinical applications. To test the cytotoxicity of bromosporine, PBMCs from HIV-negative donors were treated with bromosporine at different concentrations for 72h and cell viability was measured by CCK-8 assays. As shown in Figure 4, no marked reduction in cell viability was detected when the concentration increased from 1 μM to 10 μM; however, on treatment with concentrations >10 μM, cell viability was obviously reduced (Figure 4). We further calculated the 50% cytotoxic concentration (CC_50_) of bromosporine and found it to be 13.04 μM (Figure 4); while the active concentration of bromosporine used *in vitro* was about 2.5 μM. Furthermore, we assessed the effects of bromosporine at its active concentration on PBMCs apoptosis by flow cytometry with Annexin V and PI staining, which could recognize the early and late apoptotic cells respectively. We found that there was no significant effect on cell apoptosis induced by bromosporine compared with mock treatment (Supplementary Figure 4). These results show that bromosporine is safe at its active concentration.

**Figure 5:**
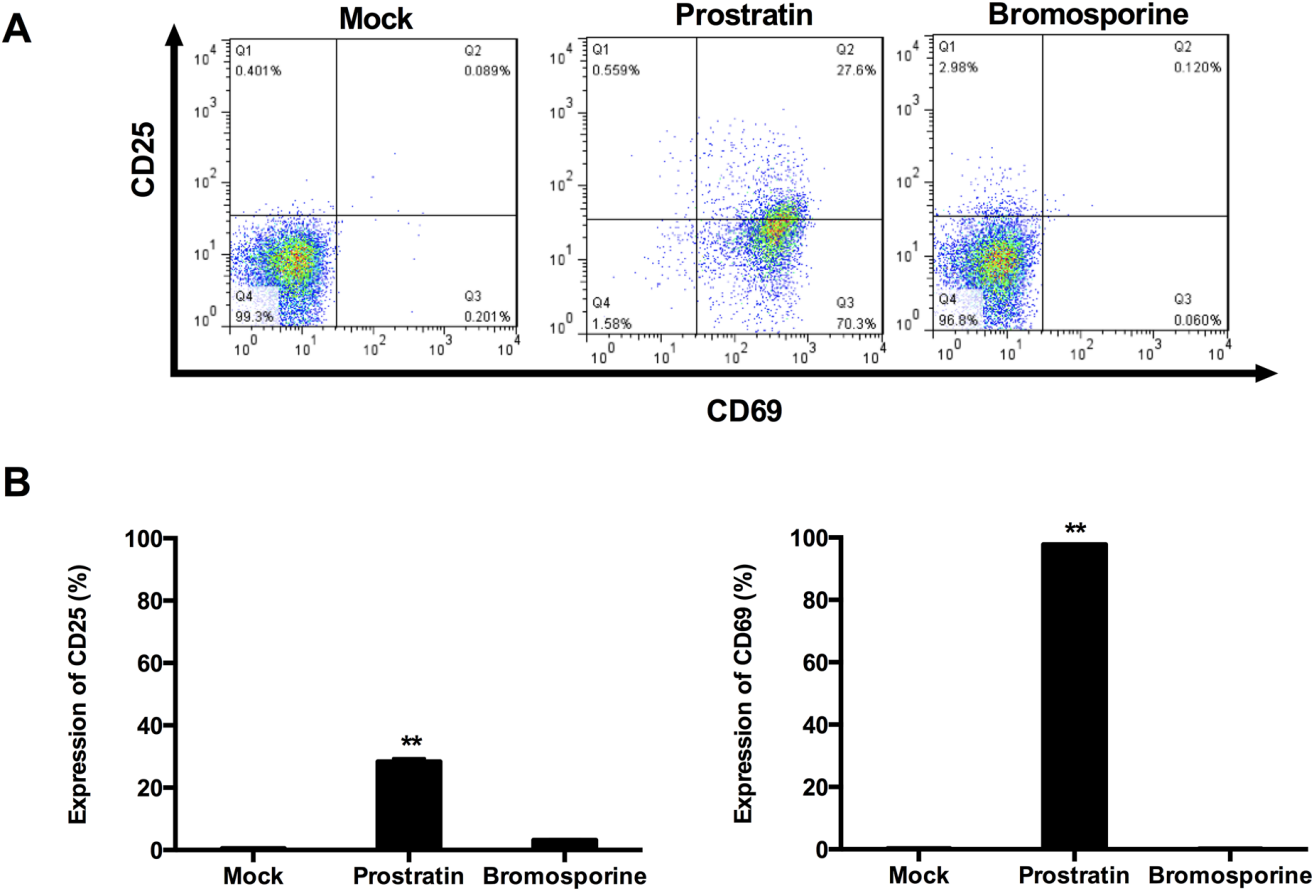
Bromosporine does not induce expression of T cell activation markers on primary CD4+ T cells **(A)** CD4+ T cells isolated from the peripheral blood of healthy HIV-negative donors were treated with prostratin (1 μM) or bromosporine (2.5 μM) for 48h and the expression of the cell activation markers CD25 and CD69 was detected by flow cytometry using specific antibodies. **(B)** Summary of the effects of bromosporine and prostratin on CD25 and CD69 expression are presented as histograms. ^*^p < 0.05, ^**^p < 0.01.

The major disadvantage of current therapeutic agents is their propensity to non-specifically activate bystander T cells [33]. To test the effects of bromosporine in T cell activation, CD4+ T cells purified from PBMCs of healthy HIV-negative donors were treated with prostratin (1 μM) or bromosporine (2.5 μM) for 48h and the expression of the T cell activation markers CD25 and CD69 was detected by flow cytometry using specific antibodies against these markers. Results showed that prostratin treatment increased the expression of CD25 and CD69 robustly relative to mock controls (Figure 5), consistent with previously reported results [34]. In contrast to prostratin, there was no significant induction of CD25 and CD69 expression observed in CD4+ T cells treated with bromosporine at its active concentration after 2 days (Figure 5). These results illustrate that bromosporine display much lower cytotoxicity than prostratin, which make it a potential activator candidate for further clinical applications.

### Tat plays a critical role in the reactivation of latent HIV-1 induced by bromosporine

**Figure 6:**
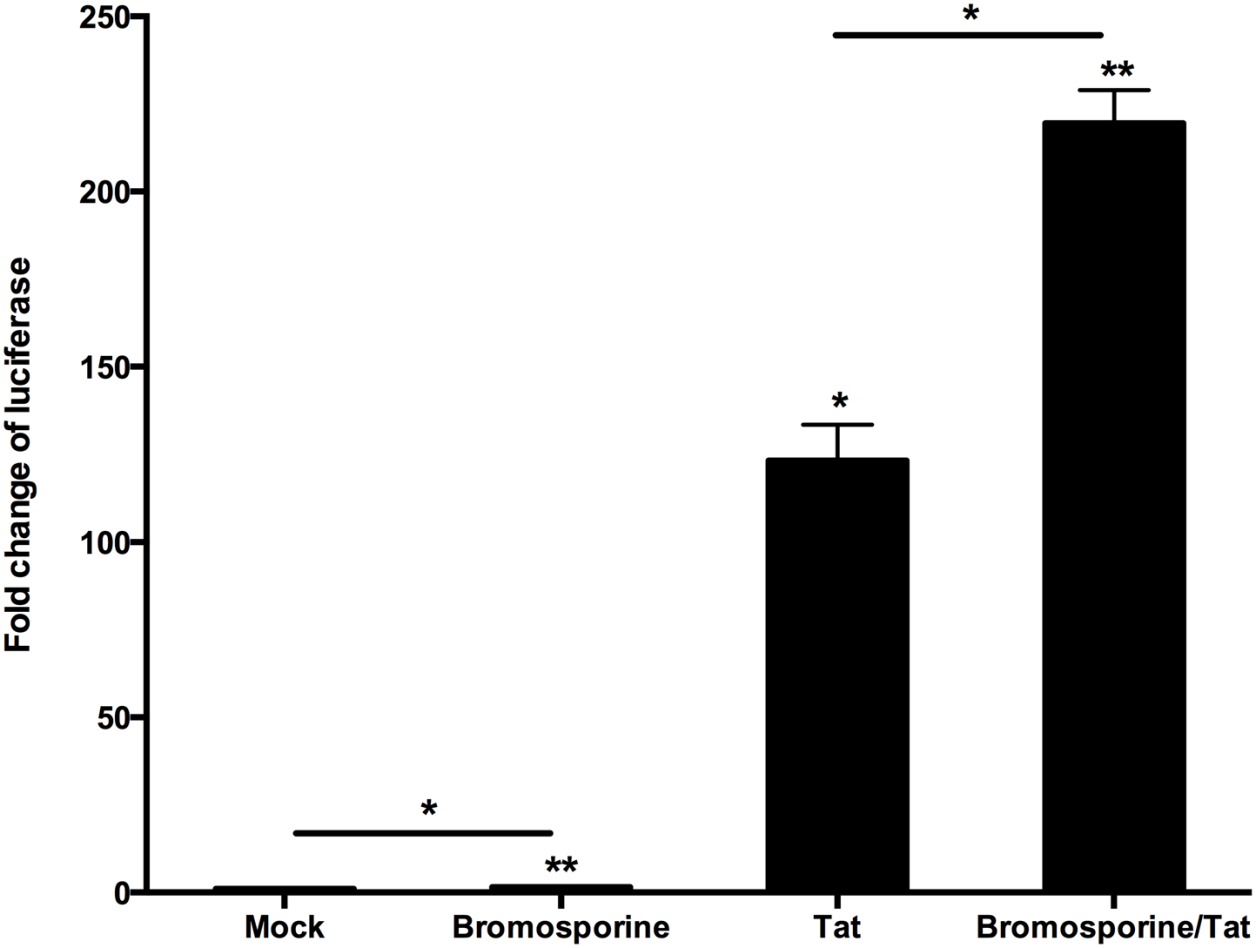
Tat plays a critical role in the reactivation of latent HIV-1 induced by bromosporine HeLa-based TZM-Bl cells isolated from the peripheral blood of healthy HIV-negative donors which contain an integrated HIV LTR-luciferase construct were nucleofected with a plasmid expressing Tat (+) or a negative control plasmid (−). Cells were then treated with bromosporine (2.5 μM, +) or DMSO (−) as indicated. Whole cell extracts were assayed for luciferase activity. ^*^p < 0.05, ^**^p < 0.01.

The fluctuation of Tat (a kind of protein encoded by HIV-1) level below the critical threshold has been proposed to play a notable role in the establishment of latency in CD4+ T cells [35]. It has been reported that Tat can recruit P-TEFb, the cellular pause relief factor, to the transactivation response element (TAR), so that Tat can be used to reverse the latency of HIV-1 by enhancing processive RNA Polymerase II (RNAP II) transcription [23, 36–39]. According to these discoveries, we hypothesized that the bromosporine-mediated activation of HIV-1 could also have involvement with Tat. In order to investigate this issue, HeLa-based TZM-Bl cells, which contain an integrated HIV LTR-luciferase construct, were nucleofected with a plasmid expressing Tat or a negative control plasmid and then treated with bromosporine (2.5 μM) or DMSO as control. The results of assays for luciferase activity showed that bromosporine alone induced only approximately 1.5-fold LTR-driven luciferase expression relative to the mock control. Nevertheless, the activation of HIV-1 LTR sharply increased up to more than 219-fold in the cases where both bromosporine and Tat existed (Figure 6). We also performed the assays only with Tat rather than bromosporine, and the luciferase expression induced by Tat only was much lower than where Tat and bromosporine were added together (Figure 6), which indicated that Tat alone could not completely bypass the effects of bromosporine to reverse HIV-1 latency though it did play a critical role.

### Bromosporine-mediated activation of HIV-1 involves the increase of CDK9 T-loop phosphorylation

**Figure 7:**
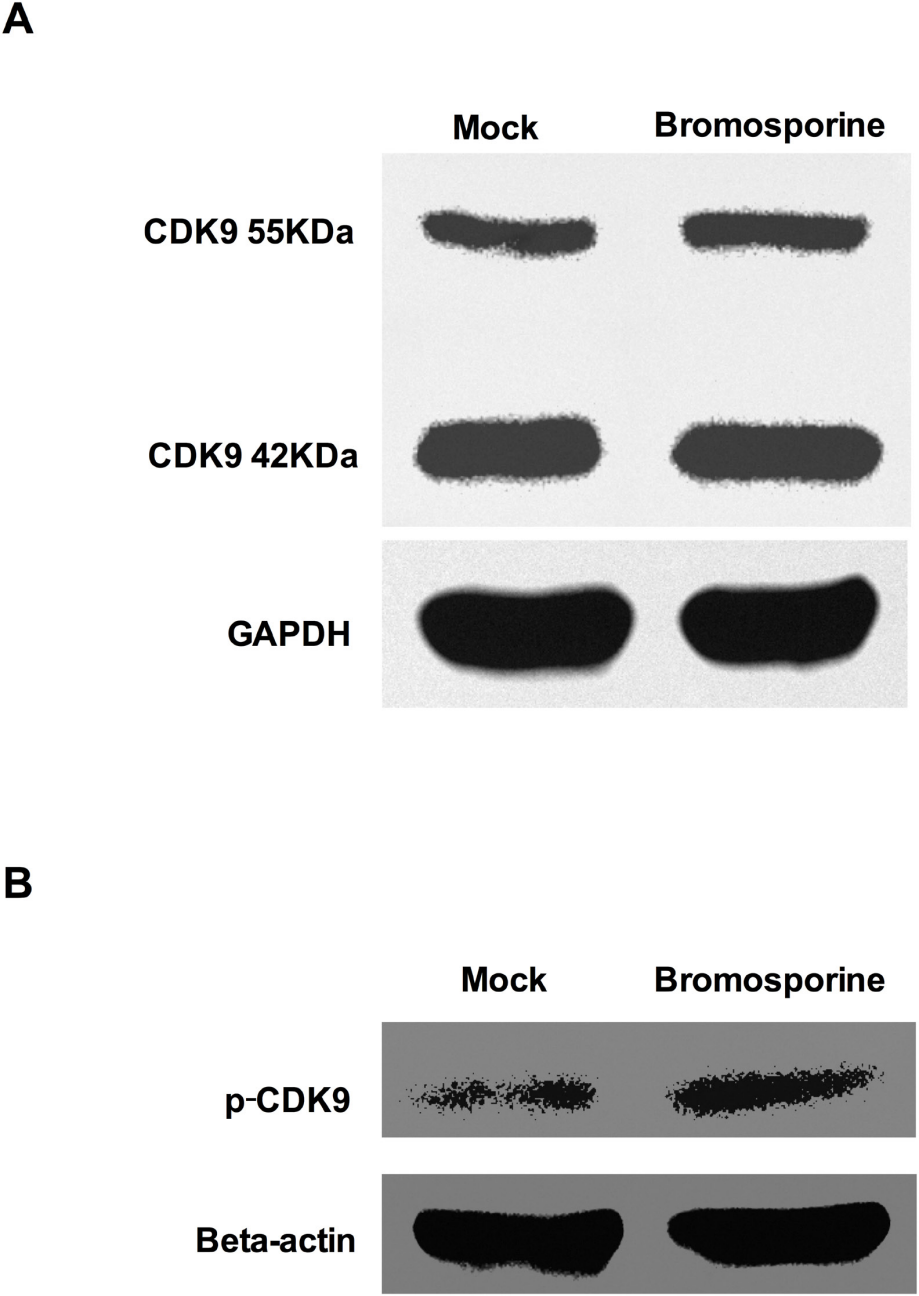
Bromosporine increases CDK9 T-loop phosphorylation in Jurkat C11 cells Jurkat C11 cells were mock-treated or treated with bromosporine (2.5 μM) for 24h, and then the levels of total CDK9 **(A)** and p-CDK9 **(B)** were determined by Western Blotting.

BET proteins can compete with Tat to bind with P-TEFb, which is composed of CDK9 and Cyclin T1 domains. BET inhibitors alter the available cellular pool of P-TEFb by obstructing the functions of BET proteins and then the phosphorylation of Thr186 in CDK9 T-loop enzymatically activate CDK9, which can phosphorylate the CTD of RNAP II and the negative elongation factors, thereby increasing the rate of HIV-1 transcription [40]. To determine whether bromosporine, as one kind of BET inhibitors, can influence P-TEFb and CDK9 T-loop phosphorylation, Jurkat C11 cells were treated with bromosporine at its active concentration and Western Blotting was performed. Results showed that the total level of CDK9 did not increase (Figure 7A). However, the prominent increase of p-CDK9 (Figure 7B) indicates that CDK9 phosphorylation was promoted.

## DISCUSSION

The major barrier to HIV-1 elimination is the persistence of latent reservoirs mostly in resting CD4+ T cells. “Shock and kill” is one of the strategies to eradicate the virus and completely cure AIDS. In this strategy, the first step is to use agents to reactivate the expression of latent HIV-1. After reactivation, the infected cells could be susceptible to the effect of ART or/and antiviral immune clearance, thereby achieving the aim of purging latently infected cells as well as protecting uninfected cells [2, 41, 42]. For this strategy, it is essential to find suitable agents both safe and able to efficiently reactivate latent HIV-1 without causing global T cell activation. Targeting different molecular mechanisms of HIV-1 latency, there have been a considerable number of compounds proposed as candidates, some of which have even entered into clinical trials [31]. However, no ideal results yet could prove any agent both safe and potent for the reactivation of latent HIV-1.

Understanding the molecular mechanisms of HIV-1 latency is the basis for researchers to seek agents for reversing the latency of HIV-1. One major rate-limiting step in the expression of HIV-1 proviral gene is the promoter-proximal pausing of initiated RNAP II [43, 44]. Whereas, the viral protein Tat can break this obstacle by direct interaction with TAR and recruiting P-TEFb to the HIV-1 LTR. P-TEFb is composed of CDK9 and Cyclin T1 domains, and enzymatically active CDK9, which requires phosphorylation of Thr186 in its T-loop, phosphorylates the CTD of RNAP II and the negative elongation factors, thereby increasing the rate of HIV-1 transcription [40]. In this signaling pathway, the BET proteins are also adverse factors for the expression of HIV-1 proviral gene. It has been reported that both BRD2 and BRD4 inhibit the activation of transcription elongation and act as an endogenous negative regulator of HIV-1 latency [21, 24]. According to the background mentioned above, BET inhibitors may be promising agents for the reactivation of HIV-1 from latency.

A novel BET inhibitor bromosporine gained our attention, and here we evaluated the ability of bromosporine to reversing latency. Using the Jurkat cells-based latency model C11, we observed that bromosporine potently reactivate HIV-1 form latency, which was shown by the dramatically increase in percentage of GFP-positive cells. Data from C11 and another Jurat cells-based latency model A10.6 cells also indicated that the anti-latency effects by bromosporine obeyed a dose- and time-dependent manner. However, the maximum GFP-positive percentage achieved with C11 cells was obviously higher than that with A10.6 cells. One possible reason for this phenomenon is the difference of the pseudovirus involved in these two cell models. The genome of J-Lat A10.6 clone does not contain the integration of a complete HIV-1 genome but the vector pEV731 [30], while the genome of C11 clone contain the vector pNL4-3 which contain the whole HIV-1 construct [28]. The second possible reason might be the different integration sites of the pseudovirus which can affect the transcription of HIV-1 [45]. Because of the variety of results in different latent models containing defective provirus, experiments with primary latent infected cells are needed for further investigation to confirm the property of bromosporine. To this end, we purified the resting CD4+ T cells from the peripheral blood of HIV-1 infected individuals on suppressive ART and treated them with bromosporine alone at appropriate concentration. The results showed that bromosporine could induce latent HIV-1 full-length transcripts effectively in those primary CD4+ T cells. Besides, most of the data showed that the effects by bromosporine were obviously better than SAHA which had been tested in clinical trials.

An important strategy most researchers have noticed is the combination therapy, like ART, which can reduce the chance of evolving drug resistance, decrease side effects and achieve enhanced potency [46]. Therefore, we investigated the synergistic activation of HIV-1 production by bromosporine with other activators. Our data showed that the effects are potently enhanced in combination with prostratin or TNF-α than using the agents alone.

To be remarkable, bromosporine displays minimal toxicity in PBMCs and primary CD4+ T cells. In our study, no remarkable impact on cell viability and cell apoptosis was detected with bromosporine at its active concentration. Furthermore, there was no significant induction of CD25 and CD69 expression observed in CD4+ T cells treated with bromosporine at its active concentration, which indicated that bromosporine did not induce global T cell activation as treatment with prostratin. It still needs further experiments to discuss the correlation between bromosporine and other types of activators.

It has been reported in several previous researches that the effect of BET inhibitors on latent HIV-1 depends on Tat in different kinds of cells like Jurkat 1G5 and HeLa-based NH1 and NH2 cells [21, 47]. In our studies, the results form Hela-based TZM-Bl cells indicate that Tat does play a critical role in the reactivation of latent HIV-1 induced by bromosporine. As for the relationship of Tat and P-TEFb, several previous researches also showed that this reactivation is P-TEFb dependent, which is involved in the catalytic activity of CDK9 [22, 26]. Our data demonstrate that bromosporine - mediated reactivation of latent HIV-1 involves the increase of CDK9 T-loop phosphorylation. Our studies provide some insights into the mechanism by which BET inhibitors work in latent HIV-1 infected cells. Nevertheless, further researches are needed to better understanding the pathway of the reactivation mediated by BET inhibitors.

In summary, we have provided strong evidence that the BET inhibitor bromosporine is a potent antagonist of HIV-1 latency and this reversal of HIV-1 latency is involved in the increase of CDK9 T-loop phosphorylation. In addition, our data also support that the bromosporine - mediated expression of HIV-1 LTR gene is highly correlated with the viral protein Tat. Those present results demonstrate that bromosporine has potential as a drug candidate in anti-HIV-1-latency therapy. Nevertheless, further studies will be necessary to extend these observations to a wider population of latent cells from infected patients undergoing ART.

## MATERIALS AND METHODS

### Cell culture

J-Lat C11 cells which were generated and used in our lab [26, 28, 48–50], were latently infected Jurkat cells encoded to express green fluorescent protein (GFP) as a marker for Tat-driven HIV long terminal repeats (LTR) expression. J-Lat A10.6 cells were obtained from NIH AIDS Reagent Program. Both the two types of cells were cultured in RPMI 1640 medium supplemented with 10% fetal bovine serum (FBS) and 1% Pen/strep at 37°C under 5 % CO_2_ humidified atmosphere. For the reactivation of HIV-1 LTR, cells were treated with bromosporine (Selleckchem), prostratin (Sigma) or TNF-α (Chemicon International).

### HIV-1 reactivation

HIV-1 reactivation was quantified by GFP expression with flow cytometry (Calibur, BD) and data was analyzed with FlowJo Software. After the treatment with bromosporine, prostratin or TNF-α, cells were washed and resuspended in PBS for flow cytometry analysis. Briefly, live cells were gated and two parameter analyses were used to differentiate GFP-associated fluorescence from background fluorescence. 10,000 gated events were acquired for each sample and the percentages of GFP-expressing cells were showed in total gated events.

### Isolation of primary CD4+ and resting CD4+ T cells

This part of experiments was performed by Shanghai Public Health Clinical Center, according to the guidelines of Bullen. et al [51]. All research participants in this study were given written informed consent. HIV-1-infected individuals were enrolled under the criteria of suppression of viremia to undetectable levels (<50 copies/ml) on ART for at least six months. Density gradient centrifugation was used for the isolation of peripheral blood mononuclear cells (PBMCs) from whole blood and then primary CD4+ T lymphocytes were enriched by negative depletion (CD4+ T cell Isolation Kit, Miltenyi Biotec) according to the manufacturer’s instructions. Resting CD4+ T lymphocytes were further enriched by depletion of CD69+, CD25+ or human leukocyte antigen DR+ (HLA-DR+) cells. The purity of the isolated resting CD4+ lymphocytes was verified by flow cytometry and was typically greater than 95%.

### Quantitative analysis for the synergism of latency-reversing agents

To evaluate the synergic activity of latency-reversing agents, the Bliss independence model was used as a metric [32]. This model is defined by the equation:

$$fa_{xy,P}=fa_{x}+fa_{y}-\left(fa_{x}\right)\left(fa_{y}\right)$$

In this equation, $fa_{xy,P}$ is the predicted fraction affected by a combination of drug x and drug y, given the experimentally observed fraction affected by treatment with drug x $\left(fa_{x}\right)$ or drug y $\left(fa_{y}\right)$ individually. The experimentally observed fraction affected by a combination of drug x and drug y $\left(fa_{xy,O}\right)$ can be compared with the predicted fraction affected, which is computed using the Bliss model $\left(fa_{xy,P}\right)$ as follows:

$$\Delta fa_{xy}=fa_{xy,O}-fa_{xy,P}$$

If $\Delta fa_{xy}>0$ with statistical significance, then the combined effect of the two drugs exceeds that predicted by the Bliss model and the drug combination displays synergy. If $\Delta fa_{xy}=0$ with statistical significance, then the drug combination follows the Bliss model for independent action. If $\Delta fa_{xy}<0$ with statistical significance, then the combined effect of the two drugs is less than that predicted by the Bliss model and the drug combination displays antagonism. In our analysis, the fraction affected was calculated as follows for the percentage of GFP-positive cells: $fa_{x}=$ %GFP-positive cells after treatment with drug x − %GFP-positive cells treated with the DMSO control.

### Measurement of intracellular HIV-1 RNA transcripts

Purified resting CD4+ T cells (5 × 10^6^) were treated with bromosporine or SAHA in the presence of 10 μM T20 and collected for RNA purification after 18h. ZR-96 Viral RNA Kit (Zymo Research) was used for extraction of total RNA. cDNA synthesis was performed by the GoScript Reverse Transcription System which contained an oligo(dT)15 primer (Promega). According to the methods reported previously [26], real-time PCR was performed in triplicate using QuantiFast SYBR Green PCR Kit (QIAGEN) on a Roche LightCycler 480 II thermocycler. Primers specific for the HIV-1 3’ polyadenylation (poly A) region were designed as described [26, 51, 52]: forward (5’ CAGATGCTGCATATAAGCAGCTG - 3’) (9501–9523), reverse (5’ TTTTTTTTTTTTTTTTTTTTTTTTGAAGCAC-3’) (9629 - poly A). Results of each drug treatment from the triplicate samples were averaged and presented as fold change relative to DMSO control.

### Measurement of cell viability and detection of T cell activation markers

PBMCs from healthy individuals were incubated with bromosporine in 96-well plates for 72h. Cell viability was evaluated using Cell Counting Kit-8 (CCK-8) (Dojindo Molecular technologies, Gaithersburg, MD, USA) as described [48]. For the measurement of changes in the cell activation status, CD4+ T cells isolated from healthy donors were incubated with prostratin or bromosporine for 48h and immunostained with anti-CD25 or anti-CD69 for 20 minutes at 4 °C. Cells were fixed in PBS and the expression of these markers was analyzed by flow cytometry.

### Apoptosis assay

After incubated with prostratin (1 μM), JQ1 (1 μM) or bromosporine (2.5 μM) for 48h, PBMCs were immunostained with FITC conjunct Annexin V and PI solution (Dojindo Molecular Technologies) for 15 minutes and subjected to flow cytometry analysis.

### Transient transfection and luciferase assays

TZM-Bl cells [53], grown in Dulbecco’s modified Eagle’s medium (DMEM) containing 10% FBS, were plated at 1 × 10^5^ cells/well in 24-well culture plates 24h before transfection and transfected with Tat or pcDNA 3.1 plasmid using Lipofectamine 2000 (Invitrogen) according to the manufacturer’s instructions. At 24h post-transfection, cells were mock-treated or treated with bromosporine. 48h after treatment, cells were lysed and Dual-Luciferase Reporter Assay Kit (Promega) was used to measure luciferase activity.

### Western blotting

Western blot analysis was performed as previously reported [49]. C11 cells were mock-treated or treated with bromosporine for 24h and lysed on ice for 30 minutes. Approximately 50–150 mg of thermally denatured protein extract was loaded on a 10% polyacrylamide gel, electroblotted onto a nitrocellulose membrane and blocked for 1h. The membrane was then incubated with CDK9 or p-CDK9 antibodies (Cell Signaling Technology). Bands were visualized using ECL Western blotting system (Santa Cruz Biotechnology).

### Statistical analysis

Means and standard errors (SE) were calculated for all data points from at least 3 independent experiments in triplicates. Statistical significance was determined using the two-way Student t test, where p value < 0.05 considered significant.

## SUPPLEMENTARY MATERIALS FIGURES



## Abbreviations

HIV: human immunodeficiency virus
AIDS: acquired immune deficiency syndrome
BET: bromodomain and extraterminal domain
TNF: tumor necrosis factor
ART: antiretroviral therapy
CDK9: cyclin-dependent protein kinase 9
HDACi: histone deacetylase inhibitor
HMT: histone methyltransferase
P-TEFb: positive transcription elongation factor b
PKC: protein kinase C
RNPS1: RNA-binding protein with serine-rich domain 1
GFP: green florescence protein
LTR: long terminal repeat
CCK-8: cell counting kit 8
PBMC: peripheral blood mononuclear cell
TAR: transactivation response element
RNAP: RNA polymerase
DMSO: dimethyl sulphoxide
CTD: C-terminal domain
BRD: bromodomain
