# Supplementary material for: The bromodomain and extraterminal domain inhibitor bromosporine synergistically reactivates latent HIV-1 in latently infected cells
Source: Oncotarget. 2017 Oct 6;8(55):94104–16. doi: 10.18632/oncotarget.21585 (PMC5706859; doi:10.18632/oncotarget.21585)
Supplement: Supplementary file 1 [file oncotarget-08-94104-s001.pdf]

The bromodomain and extraterminal domain inhibitor bromosporine synergistically reactivates latent HIV-1 in latently infected cells

SUPPLEMENTARY MATERIALS

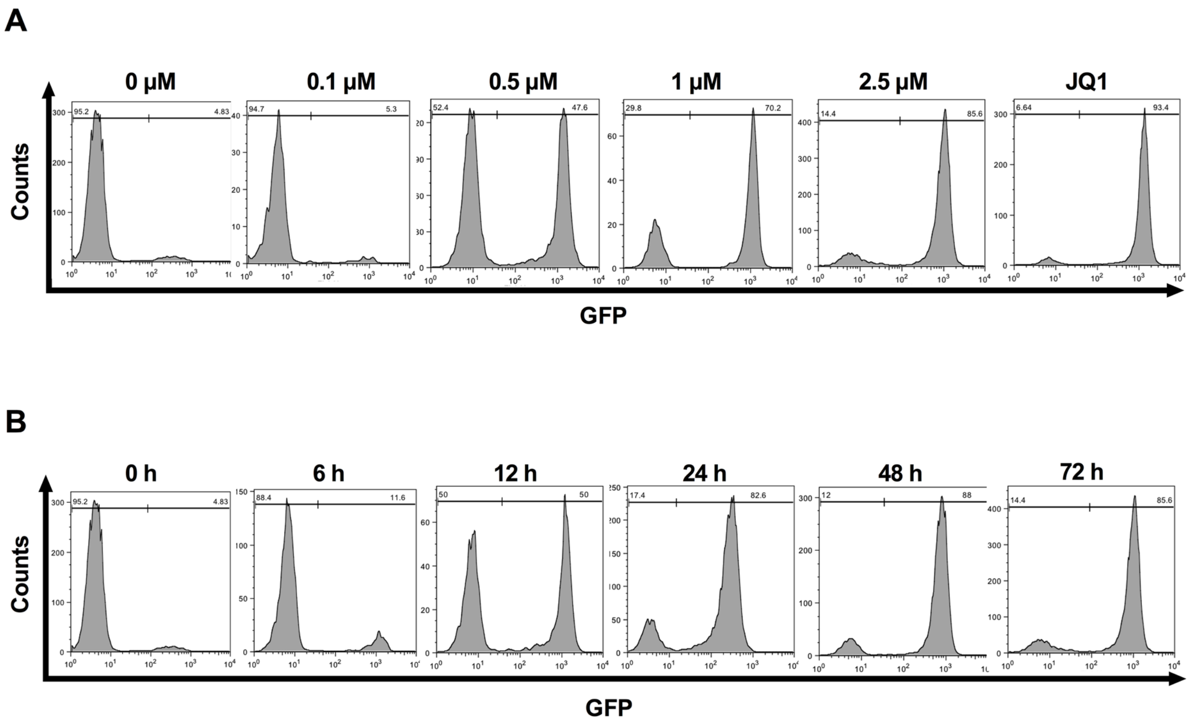

Supplementary Figure 1: Bromosporine activates HIV-1 replication *in vitro* in C11 cell model. **(A)** J-Lat clone C11 cells were treated with bromosporine for 72h at the indicated concentrations or treated with JQ1 (1 μM) for 72h and induction of GFP, representing the level of HIV-1 transcription, was measured by flow cytometry. **(B)** C11 cells were mock-treated or treated with 2.5 μM bromosporine for the indicated time period, and induction of GFP was measured by flow cytometry.

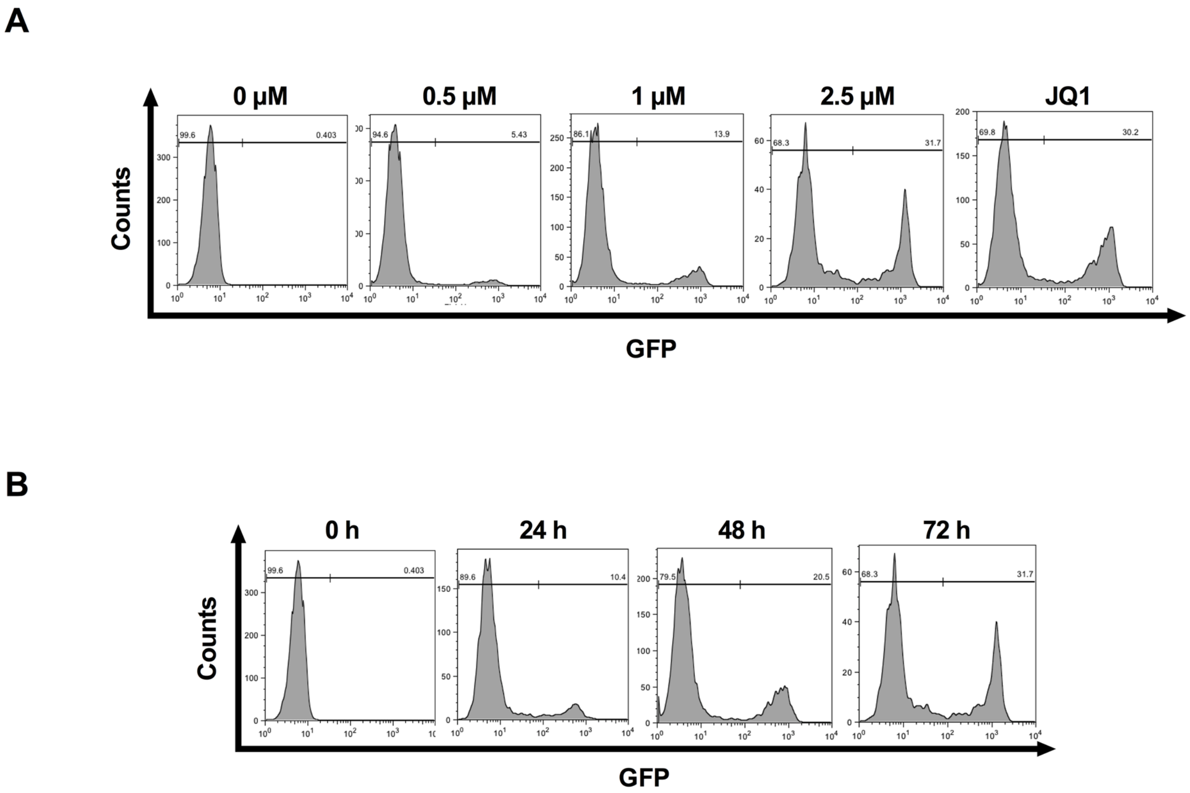

Supplementary Figure 2: Bromosporine activates HIV-1 replication *in vitro* in A10. 6 cell model. **(A)** A10.6 cells were treated with bromosporine for 72h at the indicated concentrations or treated with JQ1 (1 μM) for 72h and induction of GFP, representing the level of HIV-1 transcription, was measured by flow cytometry. **(B)** A10.6 cells were mock-treated or treated with 2.5 μM bromosporine for the indicated time period, and induction of GFP was measured by flow cytometry.

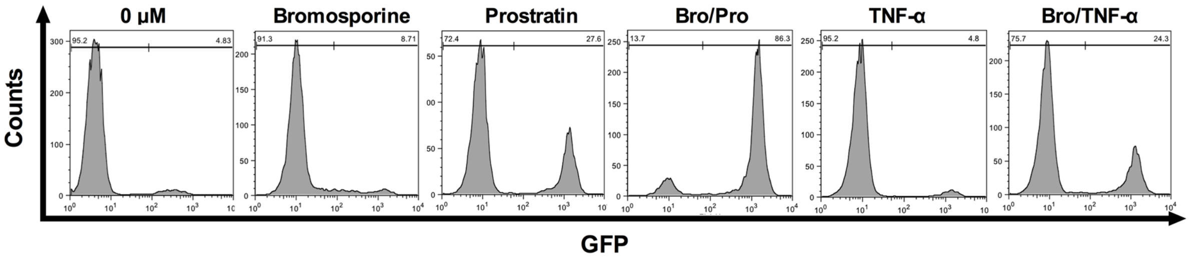

Supplementary Figure 3: Synergistic reactivation of HIV-1 promoter by bromosporine and prostratin, TNF-α in C11 cell model. C11 cells were mock-treated or treated with either bromosporine (0.25 µM), prostratin (0.2 µM), TNF-α (10 ng/µl) or bromosporine (0.25 µM)/prostratin (0.2 µM), bromosporine (0.25 µM)/ TNF-α (10 ng/µl). The effect of activation of the HIV-1 promoter was determined by quantifying GFP-positive cells 72h after treatment using flow cytometry.

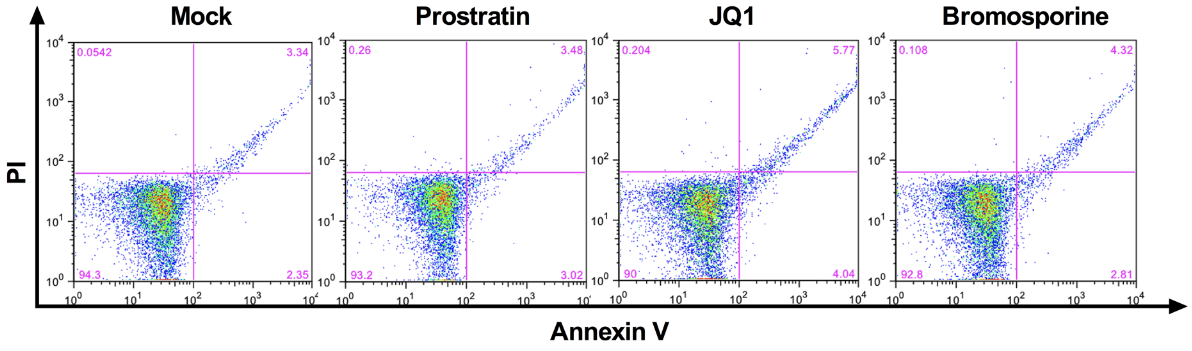

Supplementary Figure 4: The effects of bromosporine on cell apoptosis in PBMCs are non-significant at its active concentration. After incubated with prostratin (1 μM), JQ1 (1 μM) or bromosporine (2.5 μM) for 48h, PBMCs were immunostained with FITC conjunct Annexin V and PI solution (Dojindo Molecular Technologies) for 15 minutes and subjected to flow cytometry analysis.
